# RETRACTION: KIF15 Promotes Proliferation and Growth of Hepatocellular Carcinoma

**DOI:** 10.1155/ancp/9807235

**Published:** 2025-05-29

**Authors:** Analytical Cellular Pathology

RETRACTION: Y.-F. Sun, H.-L. Wu, R.-F. Shi, L. Chen, and C. Meng. “KIF15 Promotes Proliferation and Growth of Hepatocellular Carcinoma,” *Analytical Cellular Pathology* 2020, no. 1 (2020): 1–9, https://doi.org/10.1155/2020/6403012.

The above article, published online on 3 April 2020 in Wiley Online Library (wileyonlinelibrary.com), has been retracted by agreement between the journal Editor-in-Chief, Dimitrios Karamichos; and John Wiley & Sons Ltd.

The retraction has been agreed following an investigation of the concerns raised by *Hoya camphorifolia* on PubPeer [1], which identified multiple instances of figure duplication.

Specifically:

  - Figure 1b: The images representing the expression of KIF15 are identical to the images claiming to represent the expression of KIF2C in Figure 2b of [2].
  - Figure 3d: The Western Blot bands corresponding to AK4 and *β*-actin expression in the MCF7 cells have been duplicated in 2 other papers (Figure 3b in [3] and Figure 2b in [4]), where they were labelled as other proteins and cell types.
  -Figure 4a: Some of the tumors appear identical to the tumors shown in Figure 6a of [5].
  -Figure 4c: The immunohistochemistry image of Ki-67 expression in KIF15 depleted tumor tissue has been duplicated in at least 3 other publications (Figure 4c (left) in [6], Figure 5d (right) in [7], and Figure 5b (left) in [8]), while being presented as images of different tumor types.

As a result of the investigation, the data and conclusions of this article are considered unreliable.

The authors were informed of the decision to retract the paper but did not provide a response.
